# Experimental study on microstructure characteristics of saturated remolded cohesive soil during consolidation

**DOI:** 10.1038/s41598-022-23323-5

**Published:** 2022-11-01

**Authors:** Juan Zheng, Zhen Yang, Huabin Gao, Xiuying Lai, Xiao Wu, Yaolong Huang

**Affiliations:** 1 School of Civil Engineering, Putian University, Putian, 351100 China; 2 Network and Information Center, East China Jiaotong University, Nanchang, 330013 China; 3 Xiamen Iport Group Co. Ltd, Xiamen, 361000 China; 4 Taizhou Traffic Survey and Design Institute, Taizhou, 318000 China

**Keywords:** Civil engineering, Materials science

## Abstract

In this paper, drained consolidation tests of saturated remolded cohesive soil were carried out at different loading rates, and the samples at different loading stages were measured by nuclear magnetic resonance (NMR) testing. Through qualitative analysis of the relationship between the transverse relaxation time *T*_2_, peak area and porosity, the deformation micro-response of the saturated remolded cohesive soil was studied. The results showed that the transverse relaxation time *T*_2_ of the saturated remolded cohesive soil samples during the initial stage consisted of two spectral peaks, representing pores with large and small pore diameters, respectively. As loading progressed, the pore diameters in each sample group gradually shifted to smaller sizes, and the final form of the *T*_2_ spectrum was unimodal, in which the pores became denser and more uniform. In the initial stage of loading, the *T*_2_ spectrum of the soil sample with faster loading showed no obvious change trend due to the influence of drainage lag. After a sufficiently long dead load time, the final shape of the *T*_2_ spectrum was very similar regardless of loading rate, indicating that the loading rate only affected the intermediate state of the soil sample, and the final state was determined by the initial state of the sample. At the same time, after a certain dead load time, the changes in pore diameter were no longer obvious, indicating that the flowing fluid in the pore was drained and that drainage consolidation was complete. According to the positive correlation between the loading rate and maximum pore pressure, a boundary rate was obtained. We could reasonably infer that if the controlled loading rate was less than this value, drainage consolidation was complete in the main consolidation stage.

## Introduction

As a natural porous medium, the macro-mechanical properties of soil are inseparable from its complex microstructure. The microstructure of soil usually refers to the embodiment of the shape, size and various contact connections between the solid particles and pores in the soil. Many engineering examples have shown that certain macro engineering characteristics of geotechnical materials consist of the comprehensive embodiment of the readjustment and evolution of its internal elements, especially for macro deformation characteristics as determined by the changes of its micropore structure, which have always concerned geotechnical scholars.

In existing research on pore structures, the mercury intrusion method (MIP) is a relatively mature technology^1,2^. However, due to the limitations of sample size and measurement method, it results in great data distortion when used for clay materials^3^. Similarly, scanning electron microscope (SEM) analysis has a problem with the sample size, making it difficult to obtain a complete pore distribution curve^4,5^. In recent years, popular computed tomography (CT) technology can realize the dynamic, rapid, and non-destructive detection of the interior of geotechnical materials^6,7^. However, it only obtains a two-dimensional picture, and the three-dimensional spatial distribution of the internal structure must be obtained by three-dimensional reconstruction. In addition, the test accuracy is not sufficient and the cost is high. As a representative rapid nondestructive test, nuclear magnetic resonance (NMR) technology will soon occupy an important position in micromechanical testing in geotechnical engineering, as it offers highly accurate results and can detect larger sample sizes; thus, it can obtain information about all pores (including fractures) of the samples. Its results are complete and representative, and this has been verified in many studies^8–11^. Many scholars have used this technology to carry out relevant research in the field of geotechnical engineering, but most have focused on the study of rock pore structures^12–15^. Although it may be difficult to popularize NMR technology due to the complexity of soil properties and the difficulty of accurately completing the conversion from NMR relaxation time into aperture, the rapid and nondestructive testing of samples using this technology will be usefull for research on soil, especially cohesive soil. Therefore, many scholars have focused on this technology and have achieved good results^16–21^. Using NMR technology to study the critical effect of remolded loess permeability, He et al.^17^ analyzed the influence of pore size distribution on the seepage percolation of loess characteristics. Wang et al.^20^ studied the microstructure of loess samples before and after a various number of collapses under different pressures using a combination of qualitative and quantitative analyses with the assistance of NMR technology. Wang et al.^21^ also used NMR to study the cementation of microscopic aggregates of particles and the changes in the internal porosity of laterite, establishing a contact model of laterite particles that could reflect the macro-mechanical failure behavior of soil samples.

Based on existing research methods, in this work, we used NMR technology to study the microstructure characteristics of saturated remolded cohesive soil samples in different loading periods. Using the loading rate as the grouping mark of the samples, we established a relationship between the rate-dependent characteristics of deformation and the microstructure on the basis of visually describing the micro-pore response by using NMR technology, so as to provide a reference for studying the rate-dependent characteristics of deformation of cohesive soils from a micro perspective. We also provided some reference operation methods and analysis ideas for the application of nuclear magnetic resonance technology in the field of geotechnical engineering.

## Materials and methods

### Sample preparation

**Figure 1:**
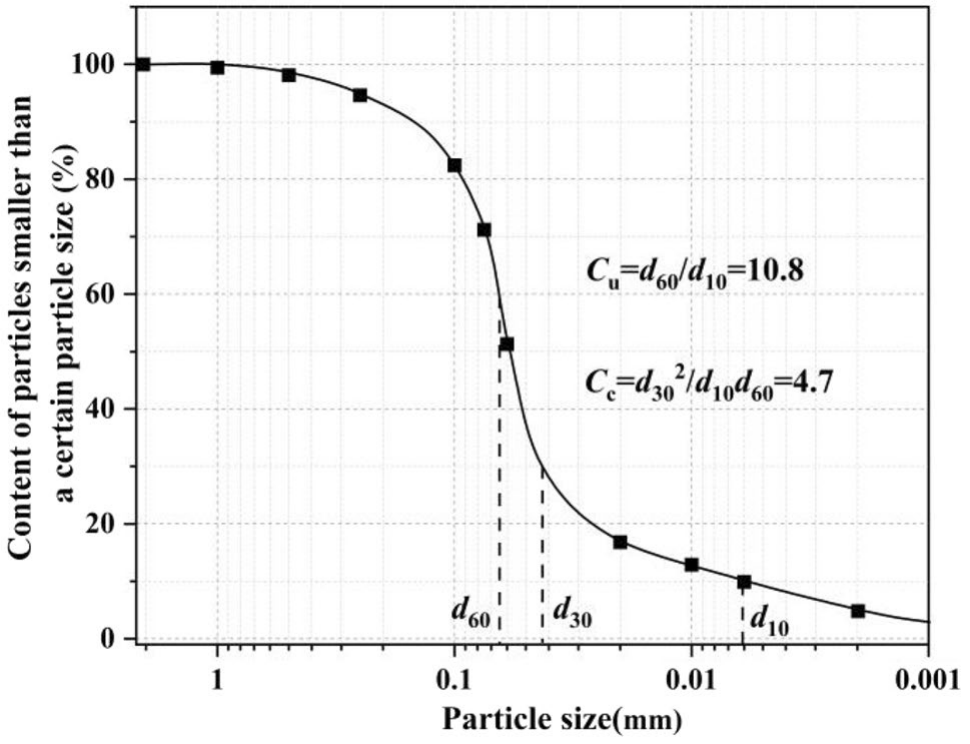
Particle grading curve of soil sample.

The soil used in this study was collected from a construction site in Liquan, Shaanxi Province, China. Its natural density was 2.03 g/cm^3^, its moisture content was 20%, and it was from a depth of about 5 m. Through simple identification, fine-grained soil with a small amount of sand particles was preliminarily determined.The retrieved soil was dried,crushed and screened to 2 mm to screen out non-soil impurities in the undisturbed soil. The particle grading curve was obtained by the particle grading analysis using the screening method and densimeter method, as shown in Fig. 1. The nonuniformity coefficient *C*u = 10.8 and the curvature coefficient *C*c = 4.7 were calculated. According to the provisions on grading in 3.1.5 of Test Methods of Soils for Highway Engineering (JTG 3430-2020), the gradation of the soil samples in this test was poor.

**Figure 2:**
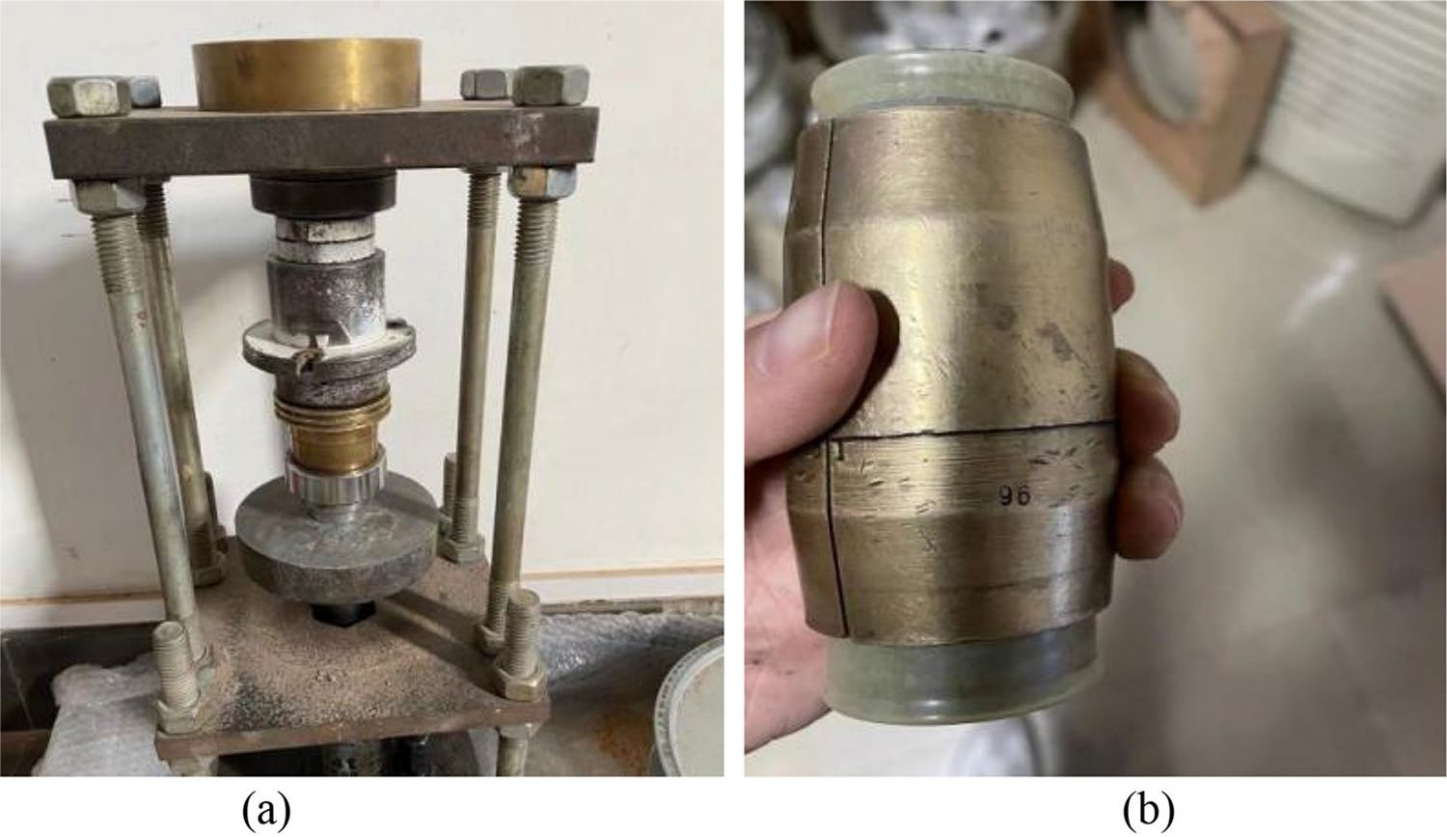
Preparation of the samples, where (**a**) shows the self-made pressing equipment, and (**b**) shows the pressed sample.

We calculated the quality of the dry soil by using the dry density, *ρ*_d_ = 1.5 g/cm^3^. After 24 h of stuffing with a moisture content value of 30%, we used self-made pressing equipment (Fig. 2) to prepare the samples in eight layers following the static pressure method. The prepared soil sample was put into the saturator and was saturated by vacuuming. The soil sample was a standard cylindrical shape with a diameter of 39.1 mm and a height of 80 mm.

### Equipment

**Figure 3:**
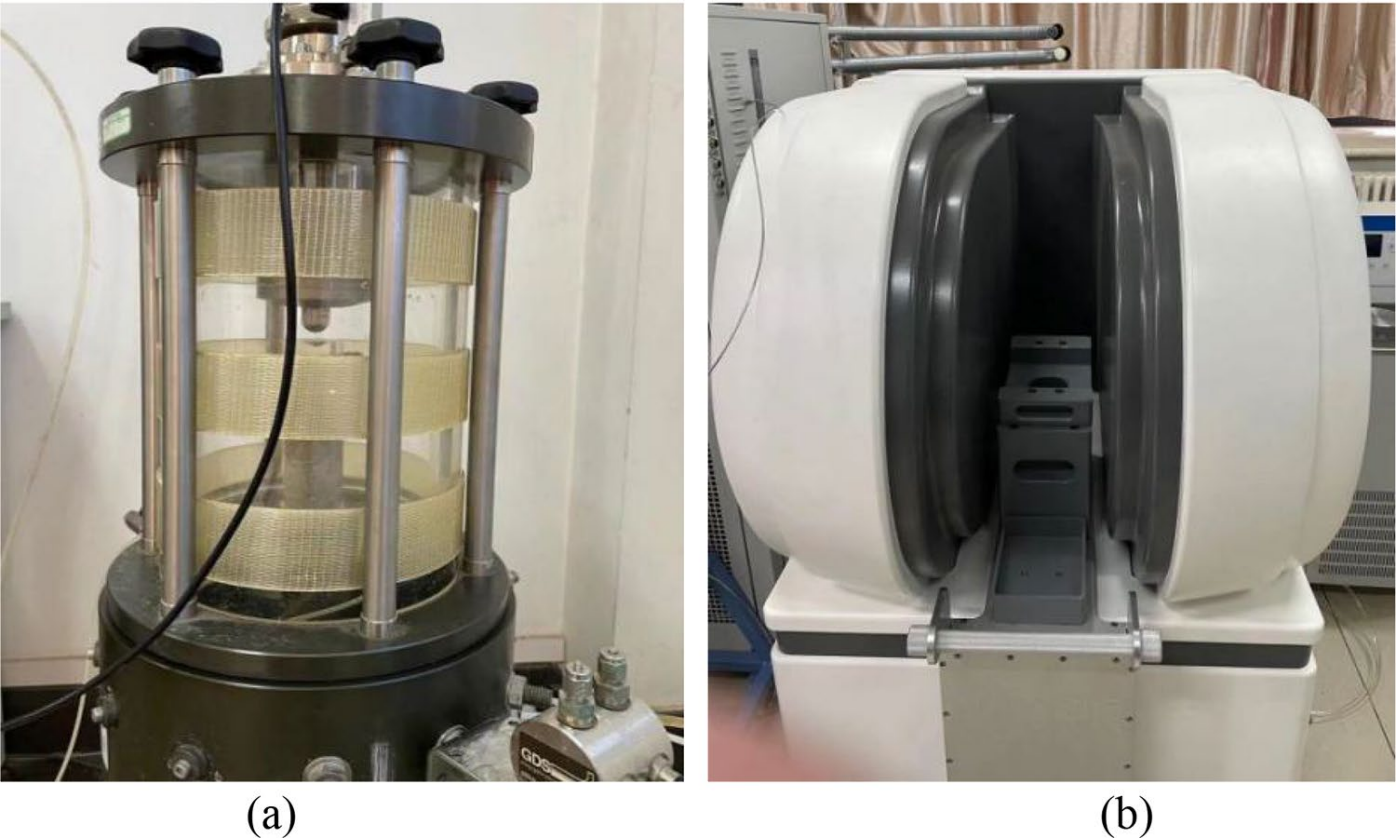
Equipment used in the test, where (**a**) shows the pressure chamber of the GDS system, and (**b**) shows the NMR system.

The test was carried out in two stages. In the first stage, a fully automatic triaxial stress-strain test system (GDS, UK) (Fig. 3a) was used to load the samples at different loading rates according to the stress-controlled mode and maintain the dead load at different times. In the second stage, the microstructures of the samples loaded in the previous stage were scanned by NMR. At this stage, a MacroMR12-150H-I nuclear magnetic resonance imaging analysis system (Newmark Electronic Technology Co. Ltd., Suzhou, China) was used (Fig. 3b). The main magnetic field of the equipment was 0.3 ± 0.05 T, the RF pulse frequency was 1.0–30 MHz, and the test temperature was 18–25 °C.

### Test design and methods

The test was carried out in two stages, and the specific steps were as follows.

• *Step* *1*: The prepared samples after vacuum saturation were preliminarily screened. The control index of screening was the quality of the saturated samples, and the error was controlled within 0.1 g.

• *Step 2*: The first stage test on the samples was carried out after the initial screening. We used GDS equipment to load the samples at different design rates to the same stress level of 400 kPa, and then maintained the dead load.

• *Step 3*: The samples loaded at the same design rate were divided into multiple samples according to the length of loading time. We took the samples with the same loading rate as a group, and used the maximum value of pore pressure *U*_max_ (or the same pore ratio) as the screening control index for the second screening. The soil samples with similar maximum values of pore pressure (or void ratio) were used as the alternative samples in the second stage of the test.

• *Step 4*: After two screenings, the samples were quickly tested by NMR (because the two devices were placed in the same laboratory, the second stage test could be completed within a few minutes after the first stage test) to obtain the transverse relaxation time *T*_2_ distribution curves.

## Test results

### Sample preparation for NMR testing

The test conducted in this study was designed into two stages. The first stage was the isobaric drainage consolidation test of saturated remolded cohesive soil at different loading rates, and the soil sample was applied to confining pressure. This stress was applied hydraulically through the sealed pressure chamber of the full-automatic triaxial stress–strain test system, loaded to 400 kPa at different rates, and maintained at a constant load of 400 kPa until the pore pressure dissipated to different degrees. The pore pressure was measured by the pore pressure sensor installed outside the pressure chamber connected with the equipment base through pipeline.The essence of the design at this stage was to prepare samples for NMR testing to assess the changes in micropore structure during the consolidation of the soil samples. The samples loaded at the same design rate were divided into multiple samples according to the length of loading time. Stop loading when the soil sample was loaded to the design value, and unloaded it through the built-in software of the full-automatic triaxial stress–strain test system. The time was set to 2 min. Next, drained the water in the pressure chamber, disassemble the soil sample, and quickly placed the soil sample with the surface treated into the NMR system for measurement.To ensure that the initial state of each sample in the sample group at the same loading rate was as consistent as possible, two sample screenings were carried out. After the samples were vacuumed and saturated, the first screening was carried out with the sample quality taken as the visual control. During the loading process, the second screening was carried out, and the pore pressure was used as the control quantity to investigate the maximum pore pressure and the variation law of the pore pressure with time. Finally, 48 groups of samples that met the screening criteria were collected.

Three groups of samples with representative loading rates were mainly selected for comparative analysis. The loading rate of the first group was 40 kPa/min, which was marked as Y1; the loading rate of the second group was 4 kPa/min, which was marked as Y2; and the loading rate of the third group was 0.8 kPa/min, which was marked as Y3. To investigate the microstructural changes of the soil samples at different stages, the samples in each group were reclassified according to the loading time.

The samples in group Y1 were divided into six samples according to no loading, loading for 5 min, loading for 10 min, loading for 10 min and dead load for 55 min, loading for 10 min and dead load for 850 min, and loading for 10 min and dead load for 1200 min; their corresponding numbers were Y1-1, Y1-2, Y1-3, Y1-4, Y1-5 and Y1-6, respectively. The samples in group Y2 were divided into six samples according to no loading, loading for 100 min, loading for 100 min and dead load for 115 min, loading for 100 min and dead load for 325 min, loading for 100 min and dead load for 830 min, loading for 100 min and dead load for 980 min; their corresponding numbers were Y2-1, Y2-2, Y2-3, Y2-4, Y2-5 and Y2-6, respectively. The samples in group Y3 were divided into six samples according to no loading, loading for 200 min, loading for 500 min, loading for 500 min and dead load for 200 min, loading for 500 min and dead load for 660 min, and loading for 500 min and dead load for 860 min; their corresponding numbers were Y3-1, Y3-2, Y3-3, Y3-4, Y3-5 and Y3-6, respectively.

**Figure 4:**
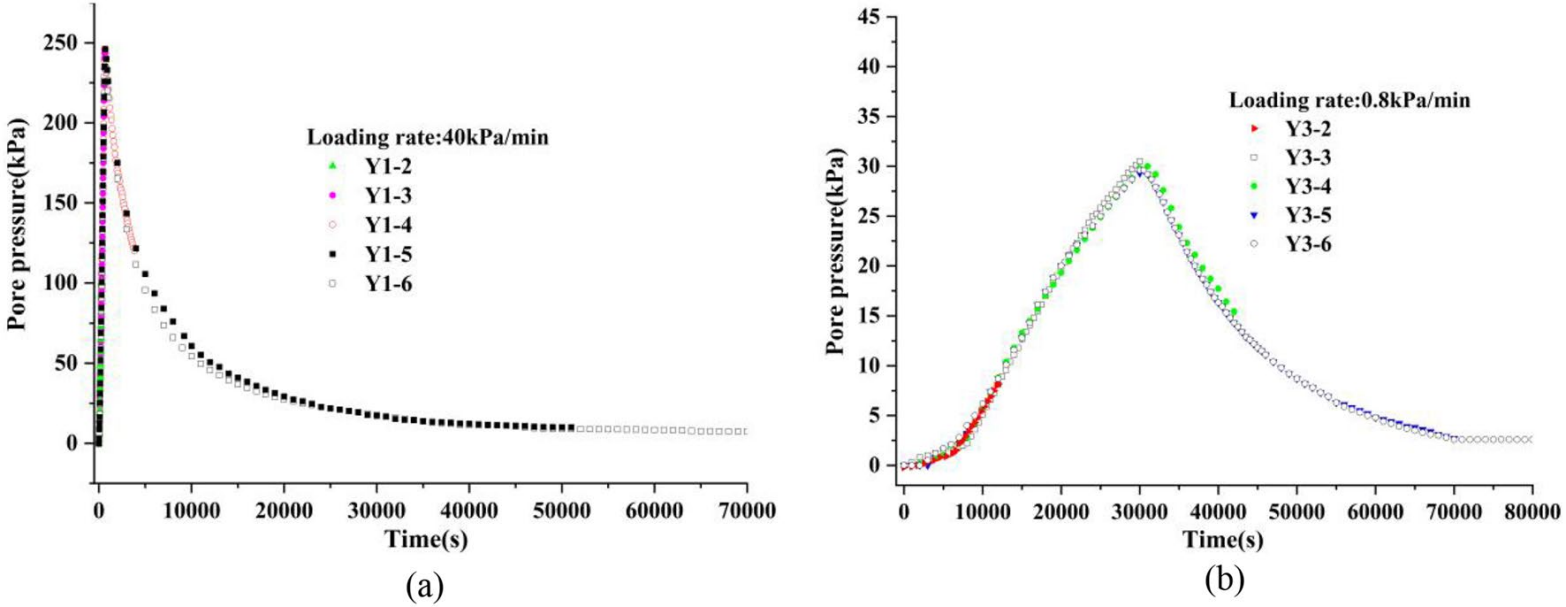
The change process of the pore pressure with time.

**Figure 5:**
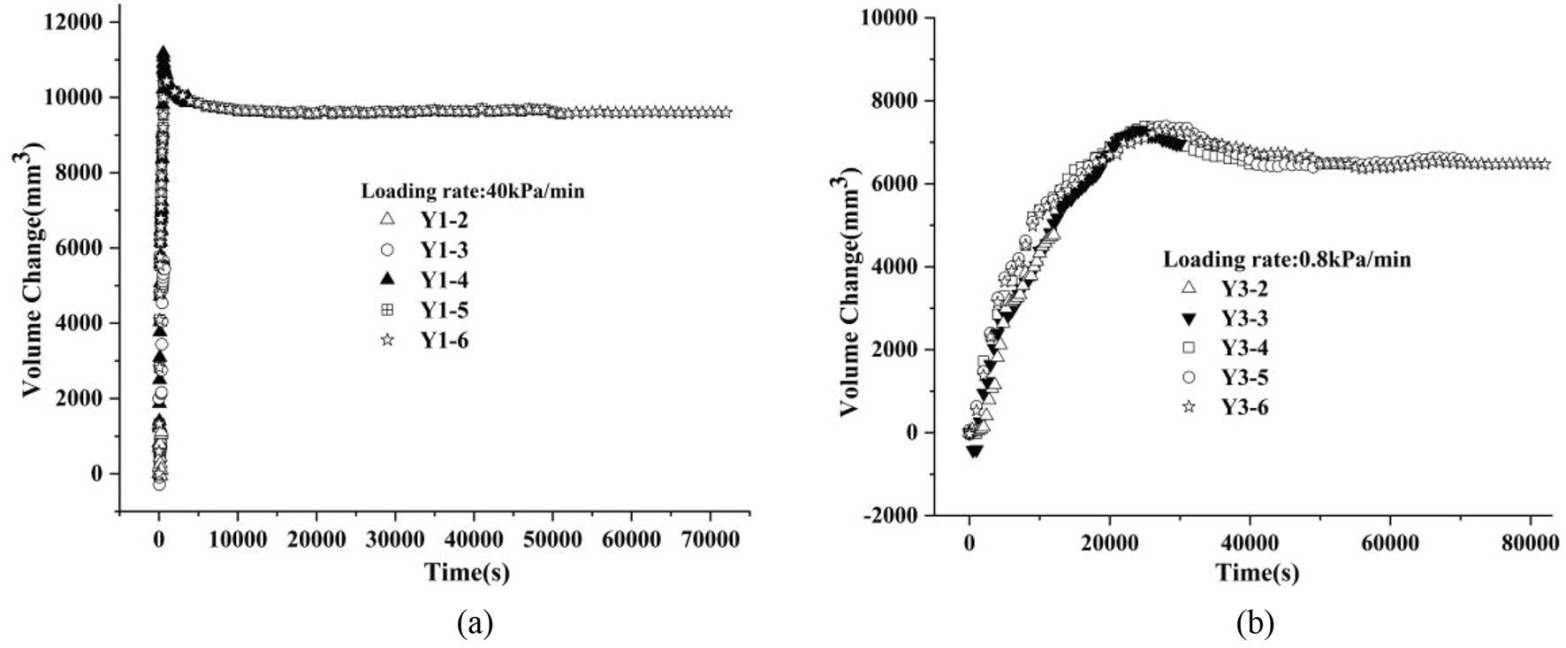
The time history of the volume change of the sample group.

Figure 4 shows the change process of the pore pressure of the sample group with time, in whith Fig. 4a is the change process of the pore pressure with time of the sample group with a loading rate 40 kPa/min, and Fig. 4b is the change process of pore pressure with time of the sample group with a loading rate 0.8 kPa/min. Figure 5 shows the history of the volume change of the sample group with time. Similarly, Fig. 5a shows the sample group with a loading rate of 40 kPa/ min, and Fig. 5b shows the sample group with a loading rate of 0.8 kPa/min.

As shown in the figure, the change path of pore pressure and volume change of each sample at different loading stages under the same loading rate was largely the same, which could reflect them having the same initial sample state.

### NMR test results

NMR is a response to radio frequency in which the nuclei in a substance become magnetized by a magnetic field; thus, relaxation is an extremely important physical quantity in NMR. The time constant of magnetization vector attenuation is called the relaxation time. The possible relaxation process could be divided into the longitudinal relaxation time *T*_1_ and transverse relaxation time *T*_2_, where the *T*_2_ value is widely used in geotechnical engineering applications^22^.

The *T*_2_ distribution reflected the pore size information, where the smaller the *T*_2_ value, the smaller the pore size. The pore size could be related to the position of the spectral peak, and the corresponding number of pores was related to the peak area. We used the *T*_2_ distribution curve of the sample group with different loading rates at different loading stages as an example.

**Figure 6:**
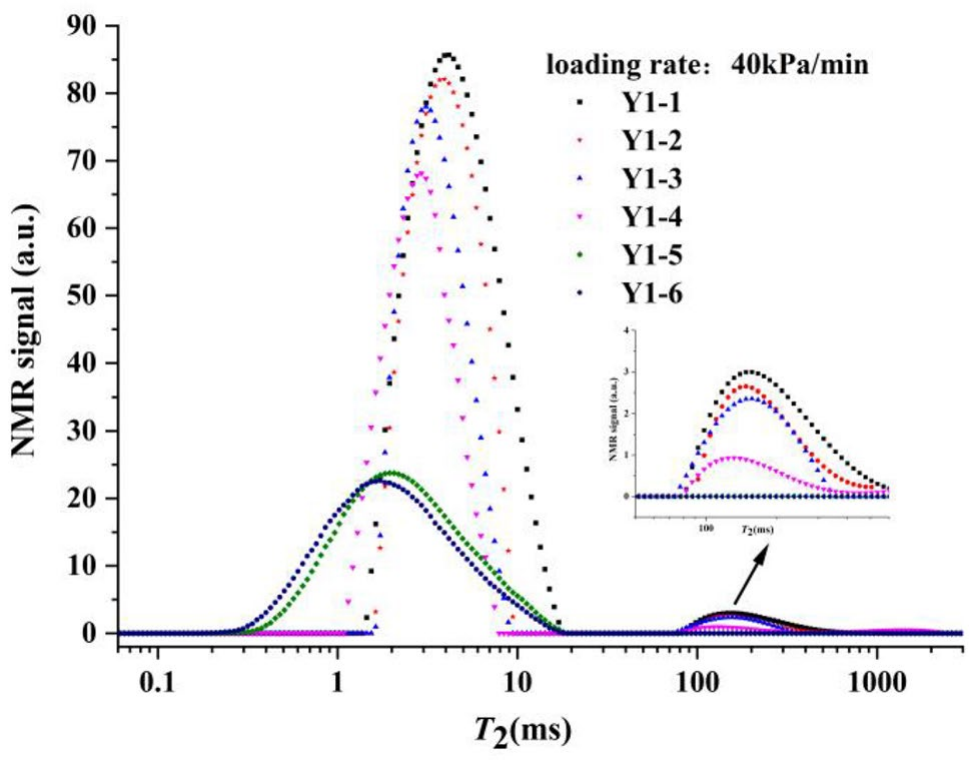
*T*_2_ distribution curve of sample group Y1 at different loading stages.

As shown in Fig. 6, the *T*_2_ spectrum curve of the initial state of this sample group was composed of two spectrum peaks. The first spectrum peak was in isolation, where the relaxation time *T*_2_ was small, and was located near 4.16 ms; it was regarded as a small aperture pore. The relaxation time *T*_2_ of the second spectral peak was large and located near 147.8 ms, and so was regarded as a large aperture pore. The great difference between the two peaks in NMR signal intensity was analyzed as the result of uneven grading of the soil samples. As loading progressed, the first peak gradually moved to the left, with a decrease in the corresponding peak. The second peak also moved slightly to the left and disappeared after sufficient time of dead load. This showed that the sample mainly contained small pores. With the continuous discharge of the pore water during the loading process, the pore volume decreased, and the relaxation time *T*_2_, which was positively related to the pore size, decreased gradually.

**Figure 7:**
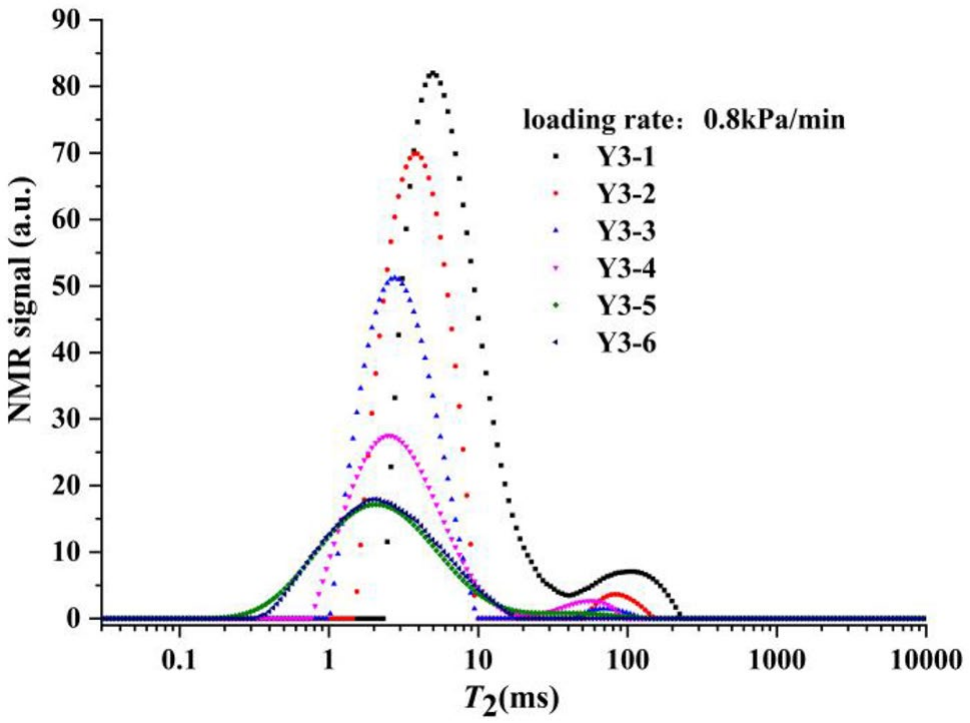
*T*_2_ distribution curve of sample group Y3 at different loading stages.

The *T*_2_ distribution curve of sample group Y3 at different loading stages is shown in Fig. 7. The *T*_2_ spectrum curve of the initial state of this sample group was also composed of two spectrum peaks, where the continuity of the pore diameter was better than sample group Y1. As loading progressed, the first peak representing the small pore diameter gradually moved to the left, and the second peak representing the large pore diameter also gradually moved to the left and finally disappeared. The overall trend was consistent with that of rapid loading.

## Analysis and discussion

### ***T***_2_ spectral area analysis of samples

According to the analysis of NMR theory, the transverse relaxation time *T*_2_ of the fluid in the pores of the porous media materials can be directly related to its internal pore structure, which can be approximately expressed as:

1 $$\frac{1}{{\mathrm{T}}_{2}}={\uprho }_{2}{(\frac{\mathrm{S}}{\mathrm{V}})}_{\mathrm{pore}},$$

where *T*_2_ is the transverse relaxation time (ms), (*S*/*V*)pore is the ratio of the pore surface area to the pore volume, (μm^2^/μm^3^), and *ρ*_2_ is the transverse surface relaxation intensity (μm/ms), which can be directly calculated according to the Schlumberger equation^23^:

2 $${\mathrm{K}}_{\mathrm{S}}={{\uprho }_{2}}^{2}{\upphi }^{4}{{\mathrm{T}}_{2\mathrm{LM}}}^{2},$$

where *K*_S_ is the saturated permeability of the soil, φ is the porosity, and *T*_2LM_ is the geometric mean value of the *T*_2_ distribution.

Although the value of *ρ*_2_ changes as the mineral composition changes, it will always be a specific value for the selected sample and will not change with shifts in temperature or pressure^24^. According to recent research^25,26^, kaolinite *ρ*_2_ = 1.8 × 10^−3^ µm/ms, glauconite *ρ*_2_ = 3.3 × 10^−3^ µm/ms, silt *ρ*_2_ = 3 × 10^−3^ µm/ms, where the range of dense sandstone is *ρ*_2_ = 12–30 × 10^−3^ μm/ms and shale *ρ*_2_ = 50 × 10^−3^ μm/ms. According to the data from Yang^27^, the *ρ*_2_ value range of Loess is *ρ*_2_ = 4.3–6.6 × 10^−3^ μm/ms. The undisturbed soil sample in this work was loess, which was reshaped after 2 mm screening. Therefore, based on the above information, the *ρ*_2_ value could be taken as 5 × 10^−3^ μm/ms.

As shown in Eq. (1), *T*_2_ is related to the specific surface of porous media and will be in direct proportion. The pores of most geotechnical media can be simplified into spherical and columnar structures, and the relationship between the specific surface and pore diameter will be *S*/*V* = *F*_S_/*r*:

3 $${\mathrm{T}}_{2}=\frac{\mathrm{r}}{{\uprho }_{2}{\mathrm{F}}_{\mathrm{S}}},$$

where *F*_S_ is the pore shape factor (for spherical pores *F*_S_ = 3; for cylindrical pores *F*_S_ = 2), and *r* is the pore radius (μm). Generally speaking, soil samples with similar *T*_2_ amplitudes will be composed of many pores with similar *S*/*V* values. If the shapes of pores differ greatly, they will be actually composed of a series of pores with different sizes. For example, the shape factors consisting of a narrow parallel plate, oblong cylindrical, and spherical pores would be 1, 2, and 3, respectively. However, if the pore shape of a sample was relatively stable, then there would be a one-to-one correspondence between *S*/*V* and *r*, and a linear relationship could be established between the relaxation rate and pore distribution. The soil sample used in this test was a reconstituted soil sample that had been screened to 2 mm. Compared with the possible impurities and natural cracks in the undisturbed soil sample, the the particle distribution of the soil sample was relatively uniform. In this study, the pore shape could be regarded as a long cylinder, thus, the shape factor was taken as 2.

As shown in Eq. (3), the *T*_2_ value was in positive proportion to the pore radius *r*, where the larger *T*_2_ value corresponded to a larger pore size. Considering this, the *T*_2_ curve of the sample could reflect the distribution characteristics of water in the pores of geotechnical media.

**Figure 8:**
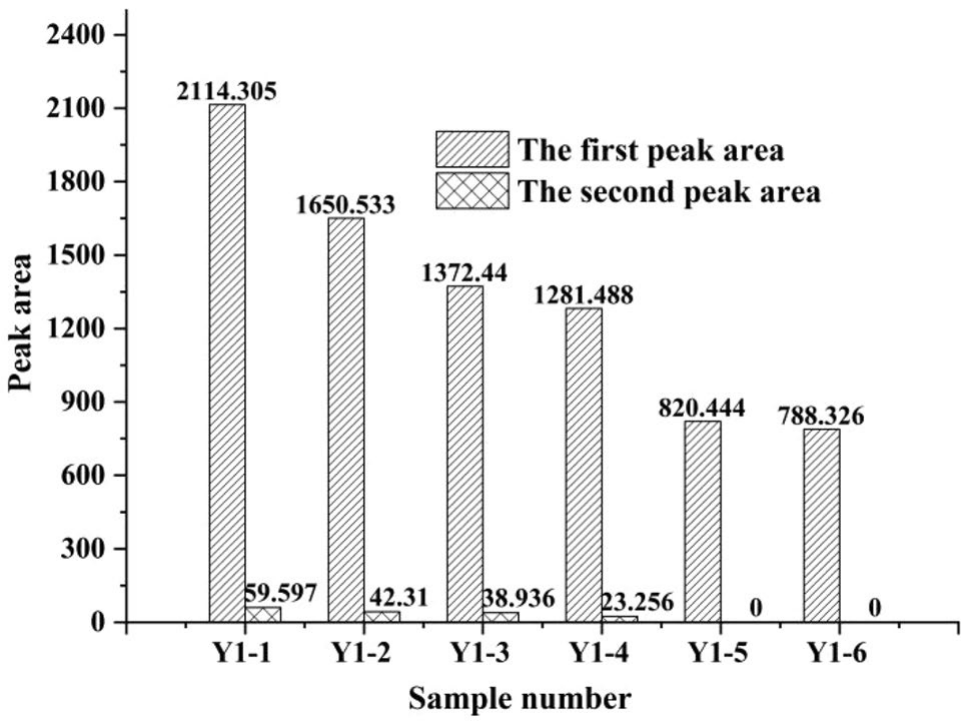
NMR spectral area distribution of sample group Y1 at different loading stages.

The *T*_2_ spectral area distribution of sample group Y1 at different loading stages is shown in Fig. 8.

We observed that the spectral areas of the samples at different loading stages in sample group Y1 gradually decreased with loading, indicating that the pore volume decreased with loading. The distribution proportion of the two peaks gradually shifted to the first peak, indicating that loading caused the larger pores to gradually drain and close, and finally become smaller.

As shown in the enlarged view of the second peak in Fig. 6 and the data marked in Fig. 8, we found that the area percentages of the two peaks did not change significantly when loaded for 5 and 10 min. The area of the second peak decreased significantly after 55 min of dead load. After 850 min of dead load, the large pores disappeared and became denser (the peak of *T*_2_ moved to the left).

**Figure 9:**
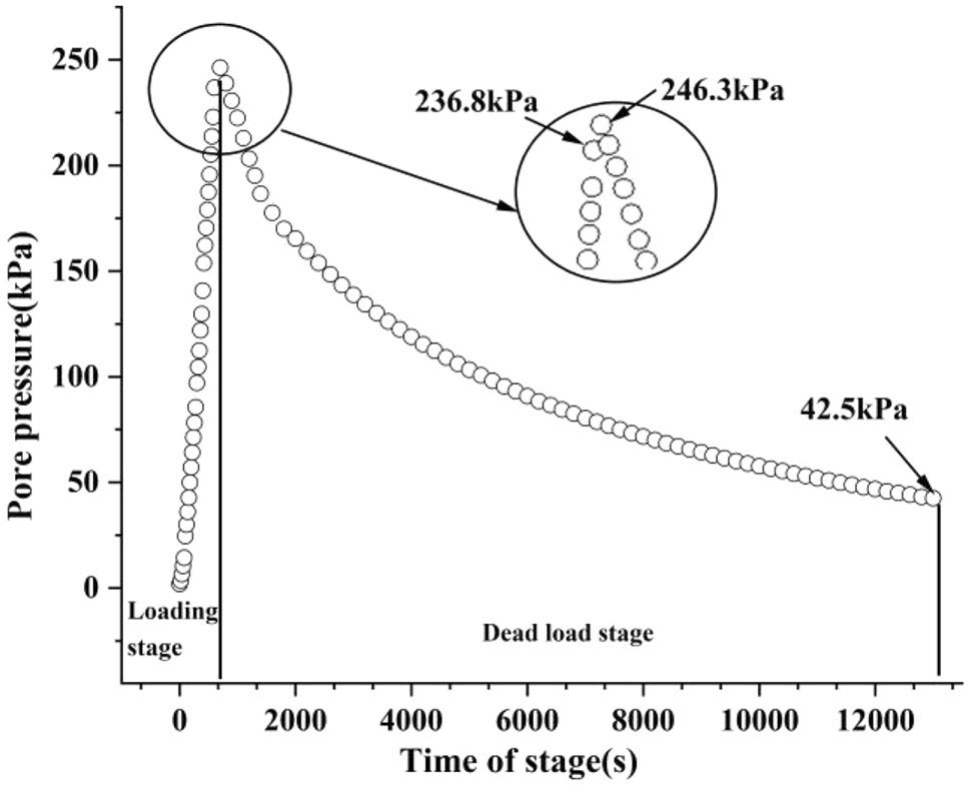
Relationship between the pore pressure and time in samples at a loading rate of 40 kPa/min.

In Fig. 9, the loading rate of the sample was 40 kPa/min. We found that the pore pressure increased sharply to 236.8 kPa after loading for 10 min, and then decreased to 42.5 kPa after dead load for 200 min. The enlarged part of Fig. 9 shows that at the end of the loading stage, the increase in pore pressure did not stop, and it reached its maximum value of 246.3 kPa at 700 s. This phenomenon was possibly caused by the drainage hysteresis: a loading rate that was too fast caused the drainage rate to be relatively slower, and the pore pressure started to dissipate after dead load. Subsequently, the drainage path was gradually became unobstructed, and finally, the two peaks converged into one peak. Thus, the large aperture pores disappeared.

**Figure 10:**
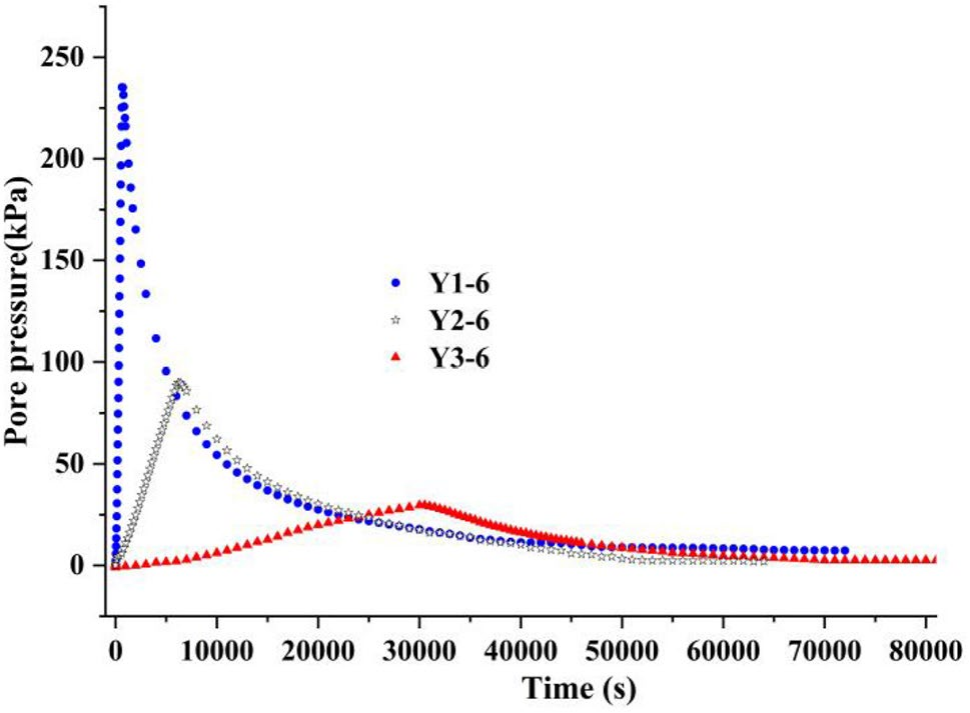
Comparison curve of the pore pressure of sample groups Y1, Y2 and Y3.

The loading rate of the sample shown in Fig. 6 was 0.8 kPa/min, which was considered slow loading in this design. When the sample was loaded to 400 kPa, the decreasing trend of the spectral area of the sample was more obvious than that in sample group Y1. According to the comparison curve of the pore pressure in sample groups Y1, Y2 and Y3 in Fig. 10, it was not difficult to observe that the pore pressure of sample group Y3 increased slowly during loading, and sample drainage was relatively smooth.

At the same time, the final shapes of sample groups Y1,Y2 and Y3 were very similar after sufficient dead load, which indicated that the speed of the loading rate only affected the intermediate state of the soil samples, and that the final state was determined by the initial state of the samples.

### Pore size distribution analysis of NMR

According to the above analysis, *ρ*_2_ could be taken as 5 × 10^−3^ μm/ms in Eq. (3) in this work, and the shape factor *F*_S_ could be taken as 2. Thus, Eq. (3) could be further simplified as:

4 $${\mathrm{T}}_{2}=\mathrm{r}/{10}^{-2},$$

As mentioned above, the moisture content in the relaxation time interval was expressed by the peak area of the curve, which could indirectly reflect the pore volume in the interval. The relaxation time distribution was converted into pore size distribution by Eq. (4), and then it was drawn into a distribution figure, which could visually describe the pore size distribution of each soil sample.

**Figure 11:**
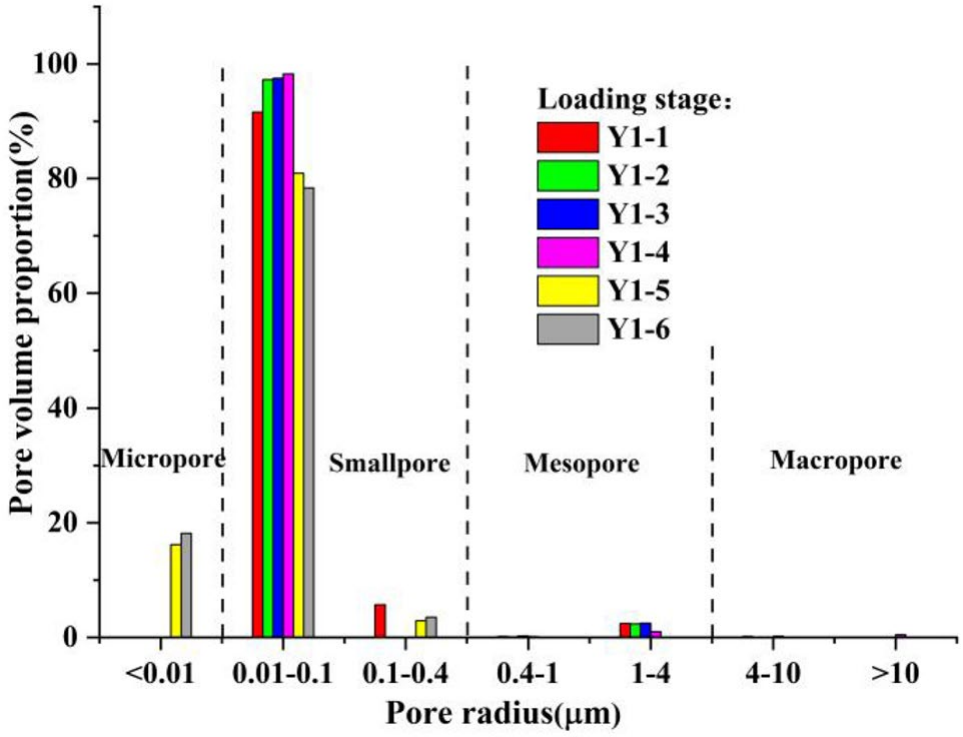
Pore size distribution of sample group Y1 at different loading stages.

**Figure 12:**
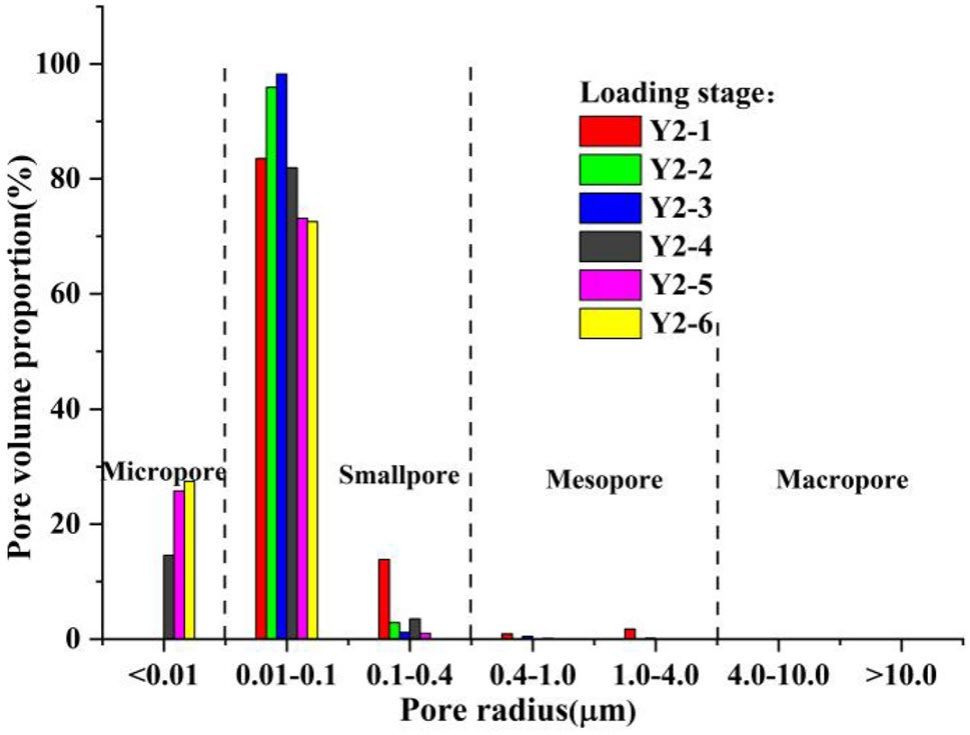
Pore size distribution of sample group Y2 at different loading stages.

**Figure 13:**
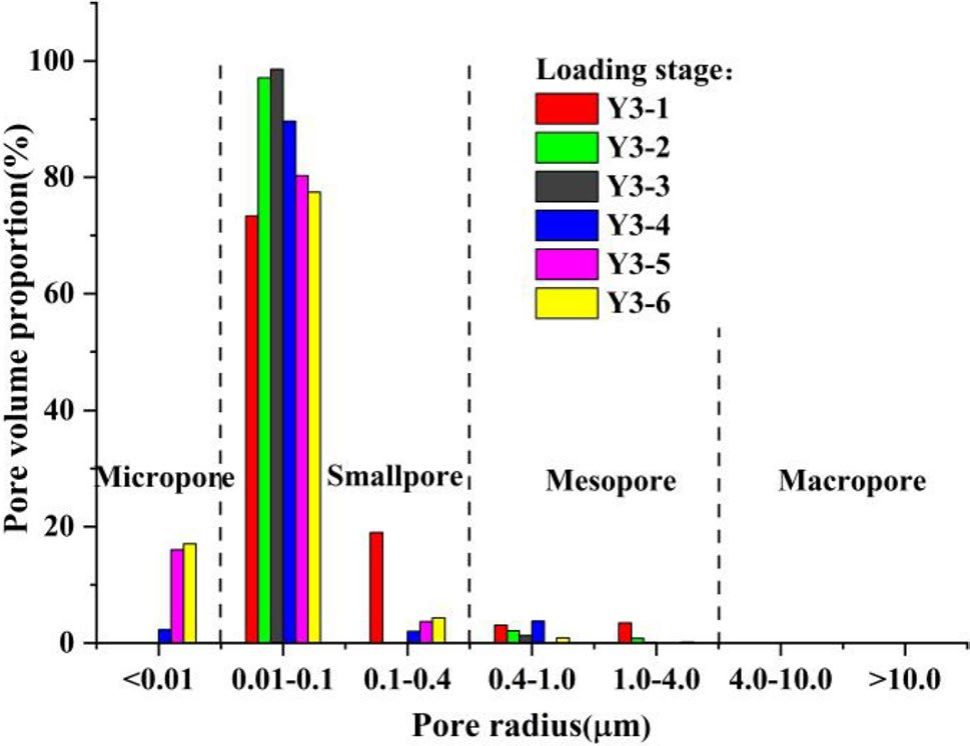
Pore size distribution of sample group Y3 at different loading stages.

The pore size distribution and comparison of soil samples under the same loading rate in the different loading stages are shown in Figs. 11–13. Among these, the pore radius r was taken as the abscissa, and the ordinate was the proportion of these pores in the total pore volume.

According to the distribution curve obtained from the NMR test and combined with the characteristics of loess, the pore radius of loess was divided into 4 categories as follows: (1) macropores with a pore radius *r* > 4 μm; (2) medium porosity with a pore radius 0.4 μm < *r* < 4 μm; (3) small pores with a pore radius 0.01 μm < *r* < 0. 4 μm; and (4) micropores with a pore radius *r* < 0.01 μm.

The sample preparation of sample group Y2 was relatively uniform, where the initial pore size distribution of the sample was concentrated in the range of 0.01–0.4 μm. This belonged to the small pore size category. During the sample preparation of sample groups Y1 and Y3, the pore radius was greater than 1 μm, making the overall trend of the three sample groups the same in the different loading stages; however, the specific range of aperture changes was different.

As shown in the figures, the pore radius values of the 3 sample groups with different loading rates were concentrated in the range of 0.01–0.4 μm at different loading stages, of which a proportion in the range of 0.01–0.1 μm accounted for the vast majority. Thus, the majority of pores were small pores, and the proportion of micropores and mesopores in the entire sample was relatively small, and there were no macropores. Compared to undisturbed loess, the pores in remolded loess were smaller, and the reason for this was closely related to sample preparation. The kneading and compaction in the process of sample preparation caused some of the soil particles to lose their structure. Specifically, the aggregation degree of the soil particles with more clay particles increased, resulting in an increase in micro and small pores in the soil.

**Figure 14:**
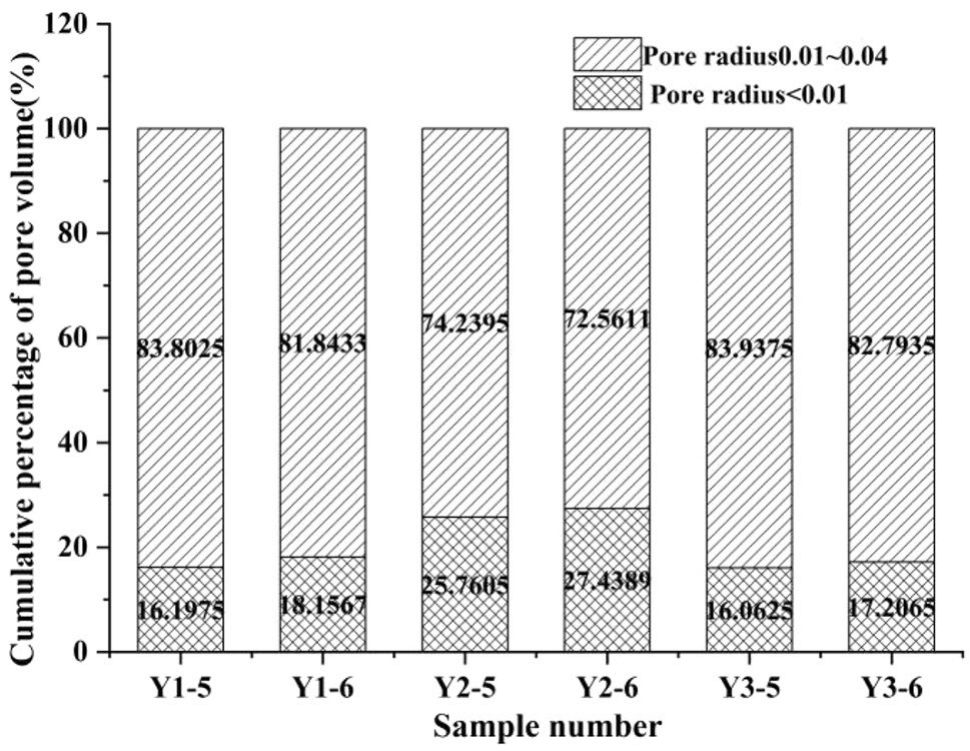
Pore distribution ratio of each sample.

As loading progressed, the pore size distribution proportion of the soil samples in each sample group gradually shifted to a smaller size. We noted that there were no micropores in the three sample groups during the loading stage, and micropores started to appear after dead load; and the longer the dead load time, the greater the proportion of micropores. At the same time, regardless of whether the loading rate was fast or slow, the pore size change was no longer obvious after dead load of a certain duration of dead load, as shown in Fig. 14.

It was clear that there was no substantial difference in the cumulative distribution ratio of the pore volume between Y1-5 (dead load to 850 min) and Y1-6 (dead load to 1200 min) in sample group Y1. The same was true of Y2-5 (dead load to 830 min) and Y2-6 (dead load to 980 min), and Y3-5 (dead load to 660 min) and Y3-6 (dead load to 860 min). Further analysis of the above data showed that this trend was closely related to the change in pore pressure.

### Discussion on creep affected by loading rate

According to the loading data of several sample groups, when the pore pressure dissipated to about 10 kPa, the pore radius would no longer decrease. This showed that the flowable fluid in the pores was exhausted; thus, the amplitude of the *T*_2_ spectrum changed very little. It was reasonable to infer that if the pore pressure did not exceed 10 kPa during the loading process to the predetermined stress value (400 kPa in this work), there was hardly any drainage in the dead load stage and the pores did not change; thus, drainage consolidation was complete in the loading stage.

**Figure 15:**
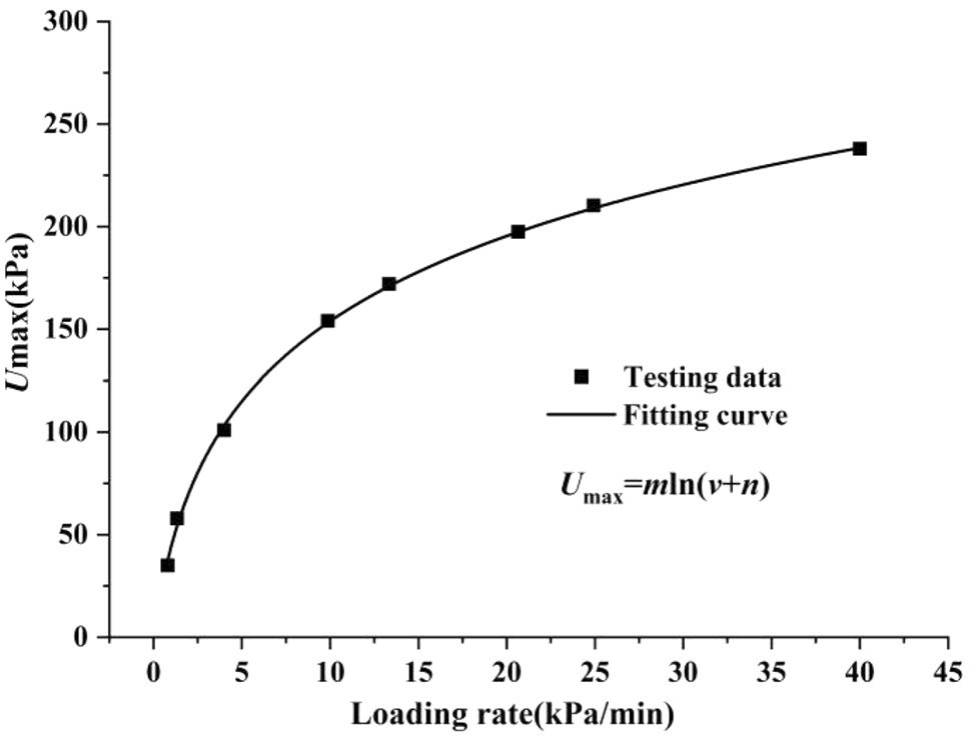
Relationship between loading rate and maximum pore pressure.

In view of the fact that the loading rate was taken as the grouping standard in this work, the relevant physical parameters of the loading rate were deliberately paid attention to when sorting out the data, and it was found that the loading rate was positively correlated with the maximum pore pressure in the loading stage, as shown in Fig. 15.

The following mathematical relationship was obtained from the trend of the loading rate and the maximum pore pressure, as shown in Fig. 15:

5 $$U_{{{\text{max}}}} = m{\text{ln}}\left( {v + n} \right),$$

where *U*_max_ is the maximum value of pore pressure, *v* is the loading rate, and *m* and *n* are the fitting parameters. In this work, *m* = 64.2 and *n* = 1. It should be noted that the maximum pore pressure value in Fig. 15 was related to the initial state of the soil sample.In this work, the quality and maximum pore pressure were used as the control standards in the sample preparation process, and the samples were screened twice to strive for consistency in the initial state. Nevertheless, for soil samples with different loading rates, the limit value of pore pressure, which made the pore diameter no longer decrease, also fluctuated up and down around 10 kPa, showing a discrete distribution.Therefore, the limit value of pore pressure mentioned above, which made the drainage consolidation completed in the loading stage, was only for the soil samples prepared in this work.

According to the above, if the maximum pore pressure of the soil sample in this test during loading did not exceed 10 kPa, the drainage consolidation was completed in the loading stage. Combining the relationship between the loading rate and the maximum pore pressure shown in Fig. 15 and Eq. (5), the loading rate corresponding to the limit value of the pore pressure can be obtained. In other words, controlling the loading rate can achieve no creep in the main consolidation stage. As mentioned above, when *U*_max_ was 10 kPa, the corresponding loading rate was only for the soil samples prepared in this work, which was the boundary rate of whether there was creep in the main consolidation stage. According to Eq. (5), when *U*_max_ = 10 kPa, then *v* = 0.17 kPa/min. In this work, if more than 2353 min was required to load to the predetermined stress value of 400 kPa, the drainage consolidation was complete in the main consolidation stage, and the stress and strain were synchronized without hysteresis, with hardly any deformation of the soil sample in the dead load stage.

Equation (5) described the relationship between the maximum pore pressure and the loading rate during the consolidation of the soil sample, and this maximum pore pressure value was obtained by testing the micro-responses of the pores by NMR test. The limit value of loading rate which controlled the creep phenomenon of soil samples in the primary consolidation stage belonged to the macroscopic physical parameter. The relationship between the two parameters is of significance for analyzing the consolidation characteristics of cohesive soil from the inside out. However, because only one kind of soil sample was used in the test, it can not be universal and can only be discussed as a phenomenon.The microstructural characteristics of different types of soil samples in the consolidation process under complex stress conditions will be my next work.

## Conclusions

In this work, we used NMR technology to study the microscopic deformation characteristics of saturated remolded cohesive soil samples under different loading rates in different loading periods.Through the analysis of transverse relaxation time *T*_2_, pore pressure, loading rate and other related physical quantities, the macro deformation characteristics were connected with the micro pore size distribution, and the rate-dependent characteristics of deformation were further analyzed, which provided a reference for studying the deformation rate-dependent characteristics of cohesive soil from a micro perspective.Our conclusions are as follows.

- (1) The transverse relaxation time *T*_2_ of the initial state of the sample consisted of two spectral peaks that represented large and small pores, respectively. As loading progressed, the pore size distribution proportion of each sample group gradually shifted to a smaller size. After a sufficiently long dead load time, the large pores gradually drained and closed, and the *T*_2_ curve became a single peak; thus, the soil sample finally became dense, with uniform small pores.
- (2) In the early stage of loading, the *T*_2_ curve of the sample group with faster loading rate showed no obvious change trend due to the influence of drainage lag. However, after a sufficiently long dead load time, the final shape of the *T*_2_ curve was very similar to the sample group with slow loading, indicating that the speed of the loading rate only affected the intermediate state of the soil sample, and that the final state was determined by the initial state of the sample.
- (3) After a certain dead load time, the pore size of the sample group with different loading rates no longer changed significantly, indicating that the flowable fluid in the pores was drained, and we considered that the drainage consolidation process was complete at this time. According to the positive correlation between the loading rate and maximum pore pressure, the boundary rate of whether there was creep in the main consolidation stage could be obtained. If the controlled loading rate was less than this rate, we could determine that the stress and strain were synchronized without lag, and that drainage consolidation was complete in the main consolidation stage.
